# Isotopologue Ratios Identify ^13^C‐Depleted Biomarkers in Environmental Samples Impacted by Methane Turnover

**DOI:** 10.1002/rcm.10118

**Published:** 2025-08-05

**Authors:** Janina Groninga, Julius Lipp, Min Song, Kai‐Uwe Hinrichs

**Affiliations:** ^1^ MARUM – Center for Marine Environmental Sciences, and Faculty of Geosciences University of Bremen Bremen Germany; ^2^ Department of Earth Sciences University of Toronto Toronto Ontario Canada

**Keywords:** compound‐specific isotope analysis (CSIA), high‐resolution‐MS, methane turnover, microbial lipids, stable carbon isotopes, UHPLC/MS

## Abstract

**Rationale:**

The stable carbon isotopic composition (δ^13^C) of individual lipids is of great value in studying carbon cycling. Among those, microbial lipids in sediments impacted by high methane turnover stand out due to their uniquely depleted isotopic fingerprint. However, gas chromatography/isotope ratio mass spectrometry (GC/irMS) is limited to volatile compounds, whereas intact polar lipids require extensive preprocessing, which results in the loss of chemotaxonomic information. Expanding compound‐specific isotopic information to intact polar lipids would enhance insights into the microbial turnover of methane.

**Methods:**

We performed ultra‐high‐performance liquid chromatography/electrospray ionization/high‐resolution mass spectrometry (UHPLC/ESI/HRMS) to analyze standards of archaeol and lipid extracts from a diverse set of sediment samples of a hydrothermal methane seep system. Using the ratio of the M1 isotopologue over the monoisotopic isotopologue M0, we calculated the δ^13^C values of archaeol and various polar, non‐GC‐amenable lipids. The δ^13^C values of archaeol obtained via ratios were compared to those measured via GC/irMS.

**Results:**

δ^13^C values of archaeol determined in natural samples via GC/irMS and the UHPLC/HRMS approach were strongly correlated (*R* ^2^ = 0.94; *N* = 76–82) across a wide range of δ^13^C values (GC‐irMS = −119‰ to −34‰). Biomarkers associated with methane turnover consistently yielded δ^13^C values below −60‰, whereas the δ^13^C values of compounds presumably associated with the photosynthesis‐based food web remained above −45‰. UHPLC/HRMS measurements of archaeol standard further indicated that δ^13^C values can be reliably determined across an M0 signal‐intensity range of approximately one order of magnitude.

**Conclusions:**

Our results highlight that the M1/M0 ratio from UHPLC/HRMS measurements can be utilized to evaluate the carbon isotopic fingerprint of non‐GC‐amenable lipids and to reliably detect lipid biomarkers putatively associated with microbial methane turnover carrying extremely depleted isotopic signatures. This paves the way for a comprehensive exploration of intact lipids associated with microbial methane turnover in environmental samples.

## 1 Introduction

The stable carbon isotopic composition (δ^13^C) of microbial lipids varies considerably due to the kinetic isotope effects of the carbon fixation pathway and the δ^13^C value of the assimilated carbon sources [1, 2, 3, 4]. Particularly distinctive isotopic signatures can be observed in microbial lipids extracted from sediments with strong microbial turnover of methane. These lipids are usually characterized by extreme^13^C‐depletion with δ^13^C values ranging from −60‰ to −140‰ [5, 6, 7, 8, 9, 10, 11]. Such negative values in these environmental settings may originate from direct incorporation of methane‐derived carbon for biosynthesis by methane‐oxidizing archaea, as suggested in initial studies that observed such strong^13^C‐depletions [5, 12]. Other possibilities are the incorporation of ^13^C‐depleted methane‐derived CO_2_ or organic intermediates by the bacterial partners [12], from chemoorganoautotrophic uptake of methane‐derived CO_2_ by anaerobic methane‐oxidizing archaea [13, 14], from heterotrophic consumption of relatively fresh biomass from anaerobic methane‐oxidizing sedimentary communities [15], or from co‐occurring autotrophic methanogens that incorporate strongly ^13^C‐depleted CO_2_, which results from the high methane‐oxidizing activity in close proximity [16]. An additional mechanism resulting in strong ^13^C‐depletion of archaeal lipids is lipid biosynthesis by certain methylotrophic methanogens [17, 18], although the presence of such products has not been confirmed in natural settings. The wide range of isotopic values of typically −60‰ to −140‰ accordingly results from high variability of δ^13^C values of methane, depending on its source and overprint by oxidative processes [19], mixed sources of the lipids from microbes with different carbon metabolisms, the rates of methane oxidation, which determine the degree of^13^C‐depletion in the porewater's inorganic carbon pool, and possibly other physiological factors. No matter what the exact mechanism is, all the abovementioned pathways leading to the accumulation of strongly ^13^C‐depleted lipids are associated with high rates of methane turnover, as typically found in seep settings, and clearly distinguish compounds associated with these processes from compounds associated with photosynthesis‐based food webs. These distinct isotopic fingerprints make compound‐specific isotope analysis (CSIA) an invaluable tool for investigating microbial methane turnover in modern and paleoenvironments.

A routine method for CSIA is gas chromatography/isotope ratio mass spectrometry (GC/irMS) [20, 21]. Although it is a powerful tool in environmental sciences, it is not without limitations. GC/irMS is mainly restricted to GC‐amenable lipids, often requiring chemical derivatization, purification, and/or cleavage reactions, constraining the available lipid pool for δ^13^C determination. More complex and unmodified biomolecules with higher chemotaxonomic specificity, such as intact polar lipids (IPLs), fall outside the scope of GC/irMS. Recently, other methods facilitating isotope analysis of intact molecules have been established in the form of spooling wire microcombustion (SWiM)‐irMS [22]. However, to obtain accurate results, SWiM‐irMS necessitates intense sample purification, which can be challenging for complex environmental samples.

During the last decade, a promising alternative technique, which shifted focus onto the isotopologue pattern derived from ultra‐high‐performance liquid chromatography high‐resolution mass spectrometry (UHPLC/HRMS), emerged in protein‐based stable isotope labeling (SIP) studies [23, 24, 25] and has recently been adopted in lipidomics [26]. By comparing the theoretical isotope pattern of a molecule with an observed pattern from stable isotope amended experiments (e.g., ^13^C, ^2^H, and ^15^N), it is possible to trace and quantify the incorporation of labeled substrates into peptides or lipids [23, 24, 25, 26]. Nevertheless, these studies exclusively focus on stable isotope‐labeled biomolecules, and the application of naturally occurring isotope patterns in environmental samples has not been fully explored because variations in natural isotope compositions are substantially smaller than those resulting from SIP experiments. However, high microbial turnover of methane in sedimentary environments is associated with a unique isotopic fingerprint that may leave a distinctive and quantifiable imprint on the isotopologues.

Recent studies have already demonstrated that high‐resolution mass spectrometry (HRMS), such as Orbitrap‐MS, operating at resolving powers > 100 000, can successfully determine the natural stable carbon isotopic composition of small organic molecules [27] such as amino acid [28] and acetate [29]. However, these HRMS‐based techniques remain limited to relatively simple analytes and are not yet applicable to more complex, intact molecules in environmental mixtures. To address this limitation, we propose an isotopologue‐based method that employs UHPLC coupled to electrospray ionization quadrupole time‐of‐flight (ESI‐QToF) mass spectrometry for CSIA of complex, chemotaxonomically valuable intact lipids. We explore to what extent δ^13^C values based on the ratio of the monoisotopic peak (M0; composed of the most abundant isotopes) and its first isotopologue (M1; one heavy isotope substituent) can differentiate ^13^C‐depleted isotopic signatures from comparatively ^13^C‐enriched compounds and lay out the conditions that must be fulfilled for reliable δ^13^C assessment. As the generally lower resolution of QToF‐MS does not allow a separation of isobaric isotopologues (^13^C vs. ^2^H), the approach focuses on large isotopic differences and offers a high‐throughput solution that balances precision, analytical feasibility, and biogeochemical insight.

## 2 Experimental

### 2.1 Sample Preparation

The sediment cores for this study were collected during the R/V Atlantis cruises AT42‐05 (Core ID: 4992‐14 and 5000‐9; November 15–29, 2018) and AT37‐06 (December 9–27, 2016) to the southern Guaymas Basin spreading center (Core ID: 4869‐3, 4870‐28, 4871‐28) and at the off‐axis Central Seep site on the northwestern Guaymas flanks (Core ID: 4867‐3) using the ALVIN submersible. The total lipid extracts (TLE) for UHPLC/HRMS and GC/irMS analysis were obtained following the modified Bligh and Dyer protocol [30] by successive extraction with solvent mixtures of dichloromethane:methanol:buffer (DCM:MeOH:buffer, 1:2:0.8, v:v:v), with the buffer consisting of a solution of monopotassium phosphate (8.7 g L^−1^, pH 7.4) for the first two extraction steps and trichloroacetic acid (50 g L^−1^, pH 2) for the final two extraction steps. The extracts were combined, washed with deionized Milli‐Q water, dried under a stream of N_2_, and stored at −20°C until analysis. An aliquot of the TLE was further processed for GC/irMS measurement by separating the *n*‐hexane‐soluble fraction into three fractions with increasing polarity (F1 + F2: hydrocarbons + ketones, F3: alcohols, F4: fatty acids) using an aminopropyl solid‐phase extraction (SPE) cartridge [12]. The fractions were eluted with 4 mL of *n*‐hexane and 6 mL of *n*‐hexane/DCM (3:1) for F1 and F2, respectively, 7 mL of DCM/acetone (9:1) for F3, and 8 mL of 2% formic acid in DCM for the final F4 fraction. The alcohol fraction containing archaeol was converted to trimethylsilyl (TMS) derivatives by treatment with *N*,*O*‐bis(trimethylsilyl)fluoroacetamide (BSTFA; Merck, Germany) and pyridine (Carl Roth, Germany) at 70°C for 1 h. Additional archaeol standards (archaeol_std_; 4ME 16:0 Diether DG; Avanti Polar Lipids, USA) were analyzed alongside the natural samples as references.

### 2.2 GC/irMS and EA/irMS

For stable carbon isotope analysis of archaeol, TMS‐derivatized aliquots of the alcohol fractions were injected into a Trace GC Ultra (ThermoFinnigan, Germany) equipped with a Restek Rxi‐5ms column (30 m × 250 μm × 0.25 μm, Restek, Bad Homburg, Germany) and coupled to a Delta V Plus irMS via a GC IsoLink and ConFlo IV interface (Thermo Fisher Scientific GmbH, Bremen, Germany). Injection was performed in splitless mode at 300°C, with a temperature program starting at 60°C for 1 min, increasing to 150°C at 10°C min^−1^, then ramping up to 320°C at a rate of 4°C min^−1^, followed by a final hold time of 17.5 min. The helium carrier gas was set to a constant flow of 1.0 mL min^−1^, and the oxidation oven was set to 940°C. The δ^13^C values were calibrated with CO_2_ reference gas (δ^13^C = −33.4‰) at the beginning and end of each run and later corrected for the three additional carbon atoms introduced by the derivatizing reagent (δ^13^C TMS = −30.1‰ ± 0.2‰). An analytical precision of < ~1‰ was determined via replicate injections of hydrocarbon standard mixtures. Additionally, a δ^13^C value of −33.3‰ ± 0.1‰ for the archaeol_std_ was determined via triplicate measurements using a ThermoFinnigan Flash 2000 Elemental Analyzer connected to a Delta V Plus isotope ratio mass spectrometer (Thermo Fisher Scientific GmbH, Bremen, Germany).

All data processing was conducted using the Isodat 3.0 software (Thermo Fisher Scientific GmbH, Bremen, Germany), with isotopic values being expressed in delta notation (δ) in parts per thousand (‰) as δ^13^C relative to the international Vienna Pee Dee Belemnite (VPDB) standard.

### 2.3 Reversed‐Phase UHPLC/ESI/MS

The TLEs from the sediment samples, as well as the archaeol_std_, were analyzed using reversed‐phase UHPLC/ESI/MS, with chromatographic separation performed on a Dionex Ultimate 3000RS UHPLC system using the separation methods of Wörmer et al. [31] (i.e., short RP) and Zhu et al. [32] (i.e., long RP).

Analyte separation, according to Wörmer et al. [31], was performed on an Acquity UPLC BEH C18 column (1.7 μm; 2.1 × 150 mm, Waters Corporation, Eschborn, Germany). The mobile phase consisted of eluent A (methanol:water, v:v, 85:15, 0.04% formic acid, 0.1% NH_4_OH) and eluent B (propan‐2‐ol:methanol, v:v, 50:50, 0.04% formic acid, 0.1% NH_4_OH), with the following gradient: 0% B for 0 min, increasing to 15% at 2 min, then to 85% by 20 min and 100% by 28 min. The flow rate was maintained at 0.4 mL min^−1^, and the column was held at 65°C.

Following Zhu et al. [32], measurements were performed on an ACE 3 C18 column (3 μm; 2.1 × 150 mm, VWR International GmbH, Darmstadt, Germany) with a mobile phase consisting of eluent A (methanol, 0.04% formic acid, 0.1% NH_4_OH) and eluent B (propan‐2‐ol, v:v, 50:50, 0.04% formic acid, 0.1% NH_4_OH). The gradient started with 0% B for 0 min, increasing to 5% by 6 min, followed by an increase to 50% and 95% by 30 and 60 min, respectively, and a final increase to 100% by 75 min. The flow rate was set to 0.2 mL min^−1^, and the column temperature was maintained at 45°C.

The RP/UHPLC system was coupled to a Bruker maXis plus ultra‐high‐resolution quadrupole time‐of‐flight mass spectrometer (UHR‐qToF, Bruker Daltonics, Bremen, Germany) equipped with an ESI interface operated in positive ion mode with a dry gas (N_2_) flow of 4 L min^−1^ and a drying gas temperature of 200°C. Spectra were acquired from 50 *m/z* to 2000 *m/z* with focus mode off, resulting in a mass resolution of 27 000 at *m/z* 1222. Every analysis was mass‐calibrated using loop injections of a calibration standard mixture (Agilent TuneMix) and further corrected using a lock mass calibrant (992.0098 Da), achieving a general mass accuracy lower than 2 ppm for all targeted compounds. All lipids mentioned were identified based on accurate mass, retention time, and characteristic fragmentation patterns in MS/MS. Extracted ion chromatograms with an isolation width of ±5 to ±10 mDa were created for the M0 and M1 peaks of all targeted lipids and integrated using DataAnalysis 5.0 (Bruker Daltonics, Bremen, Germany).

### 2.4 δ^13^C Calculation Based on M0/M1

Our approach for CSIA relies on the ratio of the M1 isotopologue and M0 peaks, calculated from the manually integrated area of both peaks in extracted ion chromatograms exported from the DataAnalysis software. The mass resolution of the QTOF‐MS is not high enough to separate isobaric isotopes that produce the same nominal M1 mass (e.g., separate ^13^C from ^2^H or ^15^N), and as a result, the M1 peak area contains isotopic contributions of several elements. To calculate the δ^13^C values from the M1/M0 ratio, we first corrected the observed M1/M0 ratio $\left(\frac{M1}{M0}\;\mathrm{obs}\right)$ by subtracting the isotopic contributions from deuterium (^2^H), oxygen (^17^O), nitrogen (^15^N), and other elements, such as sulfur (^33^S), if present, in the respective molecule, and then dividing the result by the number of carbon atoms (nC), as shown in Equation (1). The contribution of each element is determined by the number of atoms (nH, nO, and nN) present in the target compound, including atoms introduced during adduct formation, and their natural fractional isotopic abundances (^17^O = 0.00038, ^15^N = 0.00364) [33]. To ensure a more accurate ^13^C determination, we also accounted for the isotopic contribution of ^2^H by adjusting the M1/M0 based on an estimated δ^2^H value relative to the Vienna Standard Mean Ocean Water (VSMOW = 0.00015576) for target compounds.

(1) $$\frac{M1}{M0}\text{corr}=\frac{\frac{M1}{M0}\mathrm{obs}-\left(\left(\left(\frac{\delta^{2}H}{1000}+1\right)*0.00015576\right)*\mathrm{nH}\right)-\left(\mathrm{nO}*0.00038\right)-\left(\mathrm{nN}*0.00364\right)}{\mathrm{nC}}$$

The δ^2^H values of microbial lipids can vary significantly depending on environmental factors and the central metabolic pathway of their producer [34, 35, 36, 37, 38, 39, 40, 41]. Thus, we conducted a comprehensive assessment of δ^2^H variability in environmental lipids to select approximate estimated δ^2^H values that minimize the uncertainty introduced via δ^2^H variability. We compiled δ^2^H values for a wide range of lipid classes, including archaeol, biphytanes, even and odd short‐chained fatty acids, methyl‐branched fatty acids, sterols, hopanols, and phytol and its derivatives, reported from diverse environmental matrices such as marine sediments, water columns, hot springs, and lakes [34, 35, 39, 41, 42, 43, 44, 45, 46, 47, 48] (Figure S1). Lipids were categorized into their dominant biological origin (archaeal, bacterial, or eukaryotic) based on chemotaxonomy, and for each group, we determined a δ^2^H range and midpoint value. For archaeal lipids (e.g., archaeol, hydroxyarchaeol, and biphytanes), the δ^2^H values ranged from −349‰ to −118‰, with a midpoint of −234‰ and a corresponding uncertainty of ±116‰. Although δ^2^H data derived from laboratory cultures such as halophilic archaea [36], ammonia‐oxidizing archaea [49], *Sulfolobus* spp. [35], and the methanogen *Methanosarcina barkeri* [50] were not included in Figure S1, the observed range of lipid–water δ^2^H fractionation (approximately −300‰ to −150‰) is consistent with values reported from environmental matrices. Accordingly, a midpoint value of −234‰ for all δ^13^C calculations involving archaeal lipids has been utilized in this study. For lipids with a putative bacterial or eukaryotic origin, the respective values can be adapted according to Figure S1. In that regard, we note that pure culture experiments by Zhang et al. [40] and Osburn et al. [51] have shown that heterotrophic bacteria may produce lipids with significantly more ^2^H‐enriched signatures (> 50‰). However, we propose that δ^2^H values derived from environmental matrices better reflect the natural range of isotopic variability in complex mixtures. We encourage targeted adjustments regarding the estimated δ^2^H value if compound‐specific data for lipids in a given environment is available.

As demonstrated in Table 1, even a large shift in δ^2^H (i.e., ±116‰) results in a relatively minor shift of approximately ±4‰ in the corresponding δ^13^C value. This uncertainty remains minor compared to the substantial isotopic differences (> ~15‰) observed between lipids associated with methane‐derived carbon and those unrelated to methane turnover. Thus, it does not compromise our approach, which concentrates on identifying extremely ^13^C‐depleted compounds, such as those related to microbial methane oxidation [5, 6, 7, 8, 9, 10, 11], rather than permille precision. These uncertainties must be acknowledged when reporting δ^13^C values derived from isotopologue ratios, as detailed in Section 3.2 and Table 2.

**TABLE 1:** Three examples showing the influence of different δ^2^H values on δ^13^C determination using Equations (1) and (2), where the M1/M0 ratio for archaeol_std_ (C_43_H_88_O_3_ + NH_4_^+^) was adjusted using an assumed δ^2^H value of −118‰, −234‰, and −349‰ (cf. Figure S1) relative to VSMOW to account for potential variation in hydrogen isotopic composition of the lipid.

C_43_H_88_O_3_ + NH_4_^+^			Influence on calculated δ^13^C values		δ^13^C ± δ^2^H‐introduced uncertainty [‰]
M0 area	M1 area	M1/M0	δ^2^H	δ^13^C	
[arb.u.]	[arb.u.]		[‰]	[‰]	
4 308 658	2 074 659	0.482	−118	−35	−31 ± 4
			−234	−31	
			−349	−28	

**TABLE 2:** Replicate isotopologue‐based δ^13^C values of diglycosidic archaeol (2G‐AR), monoglycosidic hydroxyarchaeol (1G‐OH‐AR), and triacylglycerol (TAG)‐C48:0 calculated from UHPLC/HRMS data following the analytical method of Wörmer et al. [31]. Measurements were conducted from the surface sediments of the Cathedral Hill site (core 5000‐9, 0–2 cmbsf). δ^13^C values were calculated assuming three different δ^2^H values based on the estimated δ^2^H variability for archaeal (−234‰ ± 116‰; 2G‐AR, 1G‐OH‐AR) and eukaryotic lipids (250‰ ± 230‰, TAG‐C48:0) on the calculated δ^13^C value (cf. Figure S1). Standard deviations (SD) of replicate measurements and total propagated uncertainties were calculated using the root‐sum‐square method, combining replicate variability and δ^2^H‐related uncertainty.

	Repl.	M1/M0	δ^13^C in ‰			δ^2^H‐related shift [‰]	SD [‰] (Repl.)	SD [‰] (propagated)	Avg. δ^13^C [‰] δ^2^H = Mid.	Reported value	
			δ^2^H = Max.^a^	δ^2^H = Mid.^a^	δ^2^H = Min.^a^						
2G‐AR	#1	57.17	−109	**−106**	−103	±3	±5	±6	−105	−105‰ ± 6‰	
	#2	56.78	−116	**−112**	−109						
	#3	57.59	−102	**−99**	−96						
	#4	57.41	−105	**−102**	−99						
1G‐OH‐AR	#1	52.19	−86	**−82**	−79	±3	±5	±6	−86	−86‰ ± 6‰	
	#2	51.68	−95	**−92**	−88						
	#3	51.83	−92	**−89**	−86						
	#4	52.29	−84	**−81**	−77						
TAG‐C_48:0_	#1	57.05	−37	**−31**	−24	±6	±3	±7	−26	−28‰ ± 7‰	
	#2	57.29	−33	**−27**	−20						
	#3	57.33	−32	**−26**	−19						
	#4	57.52	−29	**−23**	−16						

^a^ 2G‐AR and 1G‐OH‐AR (both NH_4_^+^): δ^2^H: minimum: −118‰; midpoint: −234‰; maximum: −349‰ (archaeal). TAG‐C_48:0_ (NH_4_^+^): δ^2^H: Minimum: −20‰; midpoint: −250‰; maximum: −481‰ (eukaryotic) (cf. Figure 1).

Isotopic variations of nitrogen, whether introduced via adduct formation or naturally occurring in a target compound, are exceptionally small, and even exaggerated δ^15^N values (relative to atmospheric N_2_) have a negligible effect. The range of δ^15^N values observed in bulk biomass [52, 53], amino acids [52, 54, 55, 56], and nitrogen‐containing lipids [52, 57] generally remains between +30‰, representing an extremely enriched endmember, and a minimum of −30‰. NH_4_^+^ adducts, introduced via the addition of industrial NH_4_OH during UHPLC analysis, are expected to exhibit δ^15^N values close to 0‰ [58], reflecting the isotopic composition of air‐derived N_2_ from Haber–Bosch synthesis. Even when simulating a shift from +30‰ to −30‰ in nitrogen‐containing lipids, the effect on the corresponding δ^13^C value is limited to the first decimal (Table S1). With a natural abundance ten times lower than that of ^15^N, ^17^O has an even smaller effect on δ^13^C values [33].

Considering these factors, the δ^13^C value expressed in ‰ can be calculated according to Equation (2) with 0.0111802 corresponding to the^13^C/^12^C ratio of the VPDB standard.

(2) δC013=M1M0corr0.0111802−1*1000

## 3 Results and Discussion

### 3.1 Criteria for Reliable δ^13^C Determination in UHPLC/HRMS

To assess the signal stability of the M1/M0 ratio in UHPLC/HRMS, we analyzed a total of 178 LC/HRMS measurements following Wörmer et al. [31] and 105 measurements according to Zhu et al. [32], with on‐column archaeol_std_ injections ranging from 0.1 to 30 ng. Archaeol forms multiple adducts; thus, we separately calculated the δ^13^C value for the protonated form [M + H]^+^ and its ammoniated form [M + NH₄]^+^.

**FIGURE 1:**
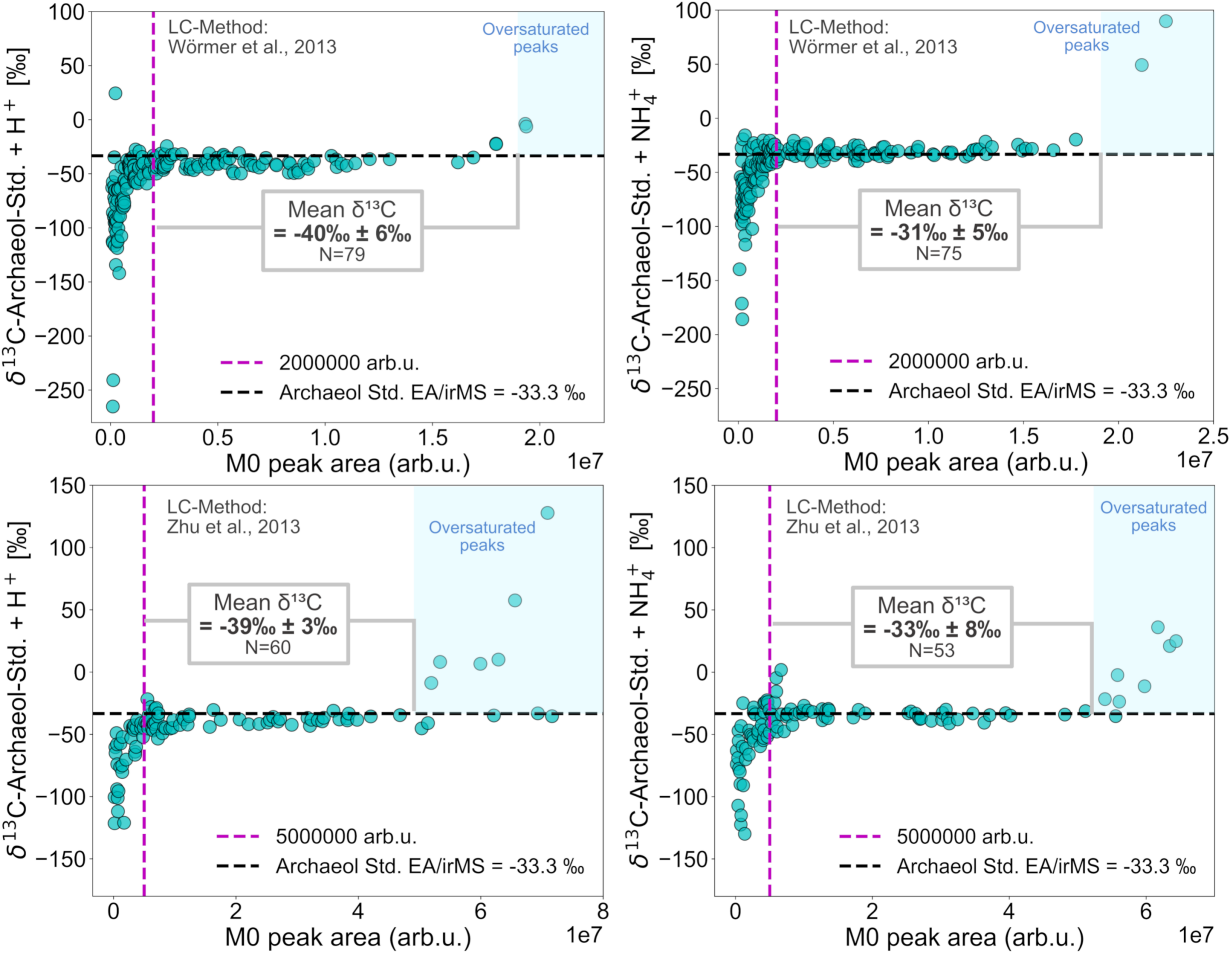
Relationship between the monoisotopic peak area (M0) in arbitrary units (arb.u.) and the calculated δ^13^C values based on Equations (1) and (2) for the archaeol standard shown. Results from the two most abundant adducts are shown, that is, archaeol + H^+^ (left panels) and archaeol + NH₄^+^ (right panels). The measurements were conducted in reversed phase‐UHPLC/ESI/qToF/MS following Wörmer et al. [31] (top panels) and Zhu et al. [32] (bottom panels), with injection amounts ranging from 0.1 to 30 ng on‐column. The area shaded in blue indicates occurrence of oversaturated M0 peaks.

Figure 1 illustrates the relationship between the M0 peak area expressed in arbitrary units (arb.u.) and the calculated δ^13^C values for the archaeol_std_ based on Equations (1) and (2), with the top panel showing data acquired from UHPLC/HRMS measurements following Wörmer et al. [31]. A key observation is the rapid decline in δ^13^C values, reaching as low as −260‰ when the M0 peak area falls below ~2 × 10^6^ arb.u., indicated by the magenta line. This clearly shows that the M1/M0 ratio becomes unreliable below this threshold. Based on the injection of archaeol_std_, on‐column amounts of 10 ng consistently yield peak areas necessary for robust δ^13^C calculation (Figure S2). It is worth noting that signal intensity generally depends on instrument performance, ionization efficiency, adduct formation, and the response factors of different lipid species. Therefore, peak areas rather than absolute quantities of the analytes provide a more robust and consistent parameter for defining thresholds.

Once the threshold is surpassed, the δ^13^C values become stable and align closely with the expected δ^13^C value of −33.3‰ determined via EA/irMS. The high signal intensity range, on the other hand, is characterized by abnormally high δ^13^C values in the upper range of the peak area. These outliers result from detector saturation, which causes an overrepresentation of the M1 isotopologue relative to M0, leading to artificially heavy apparent δ^13^C values.

A similar observation can be made when examining δ^13^C values obtained from UHPLC/HRMS measurements following Zhu et al. [32], as shown in the bottom panel of Figure 1. However, this method involves a longer chromatographic separation with a total analysis time of 90 min and lower scan rates (1 Hz) for detected compounds, resulting in generally larger peak areas. Consequently, the M0 peak area threshold for UHPLC/HRMS is higher. Based on Figure 1, we define an approximate M0 peak area threshold of 5 × 10^6^ arb.u. Once the M0 peak area threshold is surpassed, the δ^13^C values consistently remain above −45‰ and align with the expected δ^13^C value of −33.3‰ as well, and no data point falls within the range that can be considered an isotopic signature characteristic for lipids related to methane oxidation.

The relationship between δ^13^C stability and the M0 peak area for [M + NH₄]^+^ and [M + H]^+^ is comparable (Figure 1). However, a small but significant offset can be observed as the [M + H]^+^ adduct systematically yields slightly lower δ^13^C values than the expected value of −33.3‰. It is conceivable that this phenomenon results from isotopic fractionation during ionization in the ESI source, but a mechanistic explanation lies beyond the scope of this study. The isotopic composition of the adduct itself can be excluded as a cause, as its impact, even when considering extreme δ^15^N values, has minimal influence on the δ^13^C (Table S1).

For reliable δ^13^C determination, the M0 peak area must meet the following criteria (note that the absolute intensity values will differ on other instruments, and these ranges will have to be determined individually): M0 peak area above 2 × 10^6^ arb.u. for chromatographic separation following Wörmer et al. [31] and 5 × 10^6^ arb.u. following Zhu et al. [32] without signs of detector oversaturation. Furthermore, peaks showing interference from coeluting compounds must be excluded when applying the proposed method.

**FIGURE 2:**
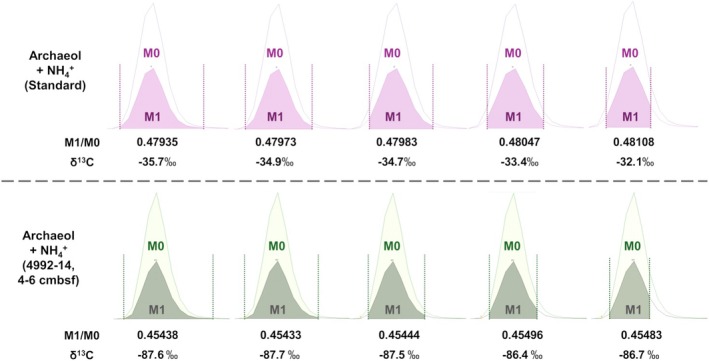
The impact of variations during manual peak integration on the δ^13^C calculation for an archaeol standard (top panel) and^13^C‐depleted archaeol from an AOM‐influenced sediment sample from the Aceto Balsamico area of Guaymas Basin (Core 4992‐14, 4–6 cmbsf) (bottom panel), both detected as [M + NH₄]^+^ adducts. Five integration approaches are shown, ranging from broader peak limits (left) to more narrowly focused on the central peak (right). Corresponding M1/M0 ratios and δ^13^C values in ‰ (Equations (1) and (2)) are displayed below each peak, with minimal variation in δ^13^C values across all different peak integration approaches.

Additionally, we studied the effect of the variability in manual peak integration on the resulting δ^13^C values by comparing five different peak integration approaches, each of which differed in the way the peak boundaries were defined (Figure 2). Overall, the results indicate that differences in peak integration only have a small effect on the calculated δ^13^C values of the target compounds, especially when the objective is the identification of extremely ^13^C‐depleted compounds.

### 3.2 δ^13^C Determination via GC/irMS vs. UHPLC/ESI/HRMS

**FIGURE 3:**
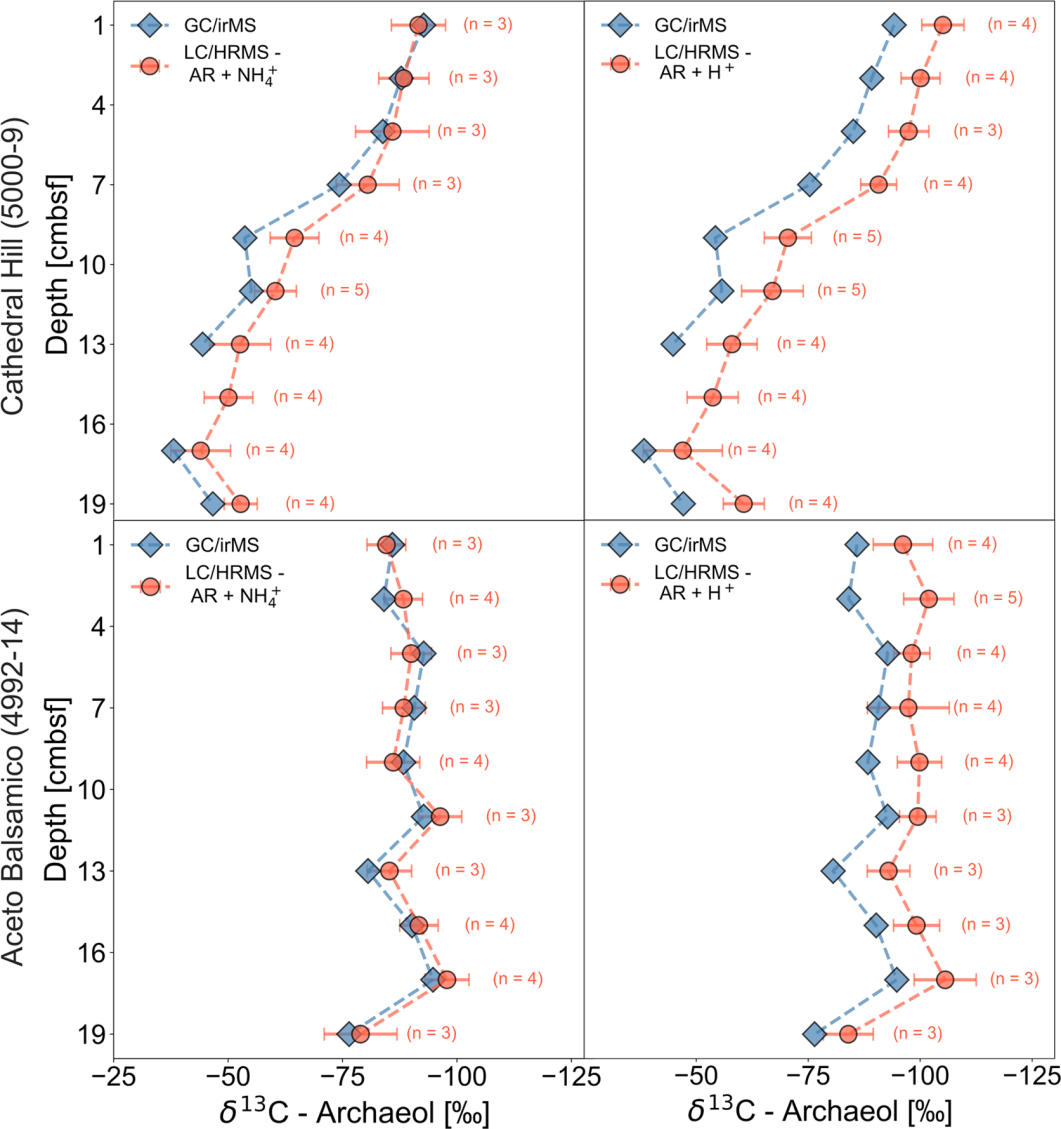
Comparison of δ^13^C values [‰] obtained from GC/irMS (blue diamonds) and reversed‐phase UHPLC/ESI/HRMS following Equations (1) and (2) for archaeol + NH₄^+^ and archaeol + H^+^ (orange circles) across two sediment cores from the Guaymas Basin. The upper panels show data from the Cathedral Hill core (5000‐9), whereas the lower panels represent samples from the Aceto Balsamico core (4992‐14). To generate δ^13^C values from the UHPLC/HRMS data, we calculated the M1/M0 ratio using the peak area of the respective peaks and applied Equations (1) and (2) using a fixed δ^2^H value of −234‰ (cf. Figure S1). Error bars reflect the propagated error, combining δ^2^H‐introduced uncertainty (±116‰) and the analytical standard deviation across multiple independent UHPLC/HRMS measurements, with *n* = X indicating the number of runs, fulfilling the following criteria: Only peaks with an integrated M0 area above 2 × 10^6^ arb.u. and 5 × 10^6^ arb.u. were included, depending on the applied LC separation methods after Wörmer et al. [31] and Zhu et al. [32], respectively (cf. Figure 1). Peaks showing signs of coelution, or oversaturation were excluded. A paired, non‐parametric Wilcoxon signed‐rank test indicated a statistically significant difference between δ^13^C values (average of replicates) derived from both adducts (*n* = 20, V = 0, *p* = 1.91 × 10^−6^) with a directional trend towards more ^13^C‐depleted values found in the H^+^ adduct of archaeol.

We compared the δ^13^C values of archaeol derived from traditional GC/irMS and those calculated from the M1/M0 ratios acquired from UHPLC/HRMS in two sediment cores collected from the hot Cathedral Hill (5000‐9) and temperate Aceto Balsamico core (4992‐14) at the Guaymas Basin (Figure 3). Both sites are well documented for the presence of anaerobic oxidation of methane (AOM) and anaerobic methane‐oxidizing archaea (ANME) [59, 60, 61, 62, 63, 64] and we have detected high abundances of archaeol in samples from both cores. Given the established link between AOM and substantial ^13^C‐depletion in the corresponding lipid biomarkers [5, 6, 7, 8, 9, 10, 11], these samples provide a robust signal for comparing δ^13^C determinations across different analytical techniques.

The δ^13^C values for the two most abundant adducts ([M + NH₄]^+^ and [M + H]^+^) were calculated independently. GC/irMS and HRMS‐derived δ^13^C profiles exhibit the same depth‐related trends in both sediment cores, particularly in core 5000‐9 (Figure 3 and Figure S3). Here, both isotope determination techniques reveal a pronounced downcore shift towards a ^13^C enrichment, highlighting that our isotopologue‐based approach captures isotopic variations. The pronounced ^13^C‐depletion of archaeol (< 70‰) in the surface sediment in both cores is consistent with the production of these compounds by ANME, and high abundances of ANME have indeed been reported in adjacent sediment cores at Cathedral Hill (5000‐11; 5000‐20) and at Aceto Balsamico (4992‐22; 4992‐23) in the form of Amplicon Sequence Variants (ASVs) [62]. Whereas δ^13^C‐methane has not been analyzed in those cores, δ^13^C‐methane values found in Guaymas Basin sediment range from −30‰ to 80‰, with more enriched values being found in strongly hydrothermally influenced settings [65, 66]. This is roughly in line with the observed offset between methane and AOM‐related lipids [12, 67]. At Cathedral Hill, the downcore increase in δ^13^C values of archaeol up to −35‰ points towards a decreased influence of ANME and a more dominant presence of other archaea, assimilating less ^13^C‐depleted carbon sources. For instance, Ramírez et al. [62] reported a heightened presence of Bathyarchaeotal ASVs in the deeper layers of the Cathedral Hill sediment, which may contribute to a more ^13^C‐enriched archaeol pool. Standard deviations in the range of ±1‰ and ±9‰, based on two to four independent UHPLC/HRMS runs, indicate good reproducibility of the δ^13^C values.

Nonetheless, a consistent offset can be observed with the UHPLC/HRMS‐based determination exhibiting slightly lower isotopic values compared to the conventional GC/irMS measurements (Figure 3). This offset can be observed for both adducts, although the [M + H]^+^ adduct consistently shows a more pronounced deviation of on average 11‰ towards more depleted δ^13^C values, in analogy to the trend observed in Figure 1. Peak intensity differences can be excluded as the cause, as both adducts were roughly equally abundant and exceeded the required M0 peak area threshold. These adduct‐specific effects should be further investigated in future studies in order to establish a comprehensive mechanistic understanding and possibly a foundation for correcting these effects. Although these effects do not perturb the differentiation between strongly^13^C‐depleted lipids characteristic of methane oxidation from those derived from other metabolic pathways, they must be acknowledged.

**FIGURE 4:**
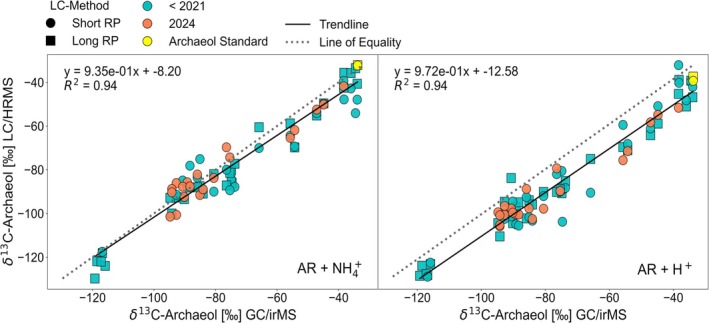
Correlation between δ^13^C values measured by GC/irMS and UHPLC/HRMS for archaeol as [M + NH₄]^+^ (left) and [M + H]^+^ (right) adducts. Squares represent UHPLC/HRMS measurements after Zhu et al. [32], whereas circles indicate data obtained following Wörmer et al. [31]. The linear regression with the corresponding equations and *R* ^2^ values, as well as the line of equality, is shown. Yellow symbols highlight δ^13^C values from an archaeol standard, green symbols represent measurements conducted before the year 2021, and orange symbols indicate repeating measurements from 2024. Only peaks with an integrated M0 area greater than 2 × 10^6^ arb.u. (LC method after Wörmer et al. [31]) and 5 × 10^6^ arb.u. (LC method after Zhu et al. [32]) were considered (see Figure 1). Peaks that showed signs of oversaturation or coelution were excluded. The total number of data points is *N* = 76 for [M + NH₄]^+^ and *N* = 82 for [M + H]^+^.

We further reinforced these findings by incorporating results from an additional sample set. Collectively, the dataset captures a wide range of δ^13^C values for archaeol in four additional sediment cores across hydrothermal vent and methane seep sites at the Guaymas Basin [68], spanning δ^13^C values from −130‰ to −34‰. As outlined in Song et al. [65] and Teske et al. [66], all sites are influenced by AOM to varying degrees. The most depleted δ^13^C values (< −110‰) were observed at the cold seep site “Octopus Mound” (Core 4867‐3) (Figure 4), consistent with admixtures of biogenic methane (−55‰ to −75‰) at this location [65, 66]. δ^13^C values acquired from the traditional GC/irMS and UHPLC/HRMS, with both adducts analyzed separately, are strongly correlated across a wide range of δ^13^C values (Figure 4). Consistent with the observation in the downcore profiles (Figure 3), the negative isotopic offset is more pronounced for the protonated molecular ions ([M + H]^+^) compared to their ammoniated counterpart ([M + NH₄]^+^).

Given the strong correlation with well‐established GC/irMS‐derived **δ** ^**13**^ **C** values and the matching depth trends illustrated in Figure 3, we propose that the M1/M0 ratio derived from UHPLC/HRMS analysis represents a promising tool for the identification of strongly^13^C‐depleted lipids and other metabolites in environmental samples typically associated with the assimilation of methane‐derived carbon, provided that the criteria outlined in 3.1 are met.

In Table 2, we provide a general guideline for reporting the analytical and δ^2^H‐introduced uncertainty of derived δ^13^C values. We performed replicate δ^13^C calculations for three selected lipids from sediment core 5000‐9 at 0–2 cm below seafloor (cmbsf). The δ^13^C values were calculated based on the range and midpoint δ^2^H values emphasized in Section 2.4 and Figure S1. The total propagated uncertainty was determined using the root‐sum‐square (RSS) method, combining the standard deviation (SD) across the replicates and the δ^2^H‐based uncertainty. The reported δ^13^C values should be accompanied by a total propagated SD, accounting for both replicate variability and δ^2^H‐related uncertainty.

### 3.3 δ^13^C Profiling of AOM and Non‐AOM‐Related Biomarkers in Hydrothermal Sediment

**FIGURE 5:**
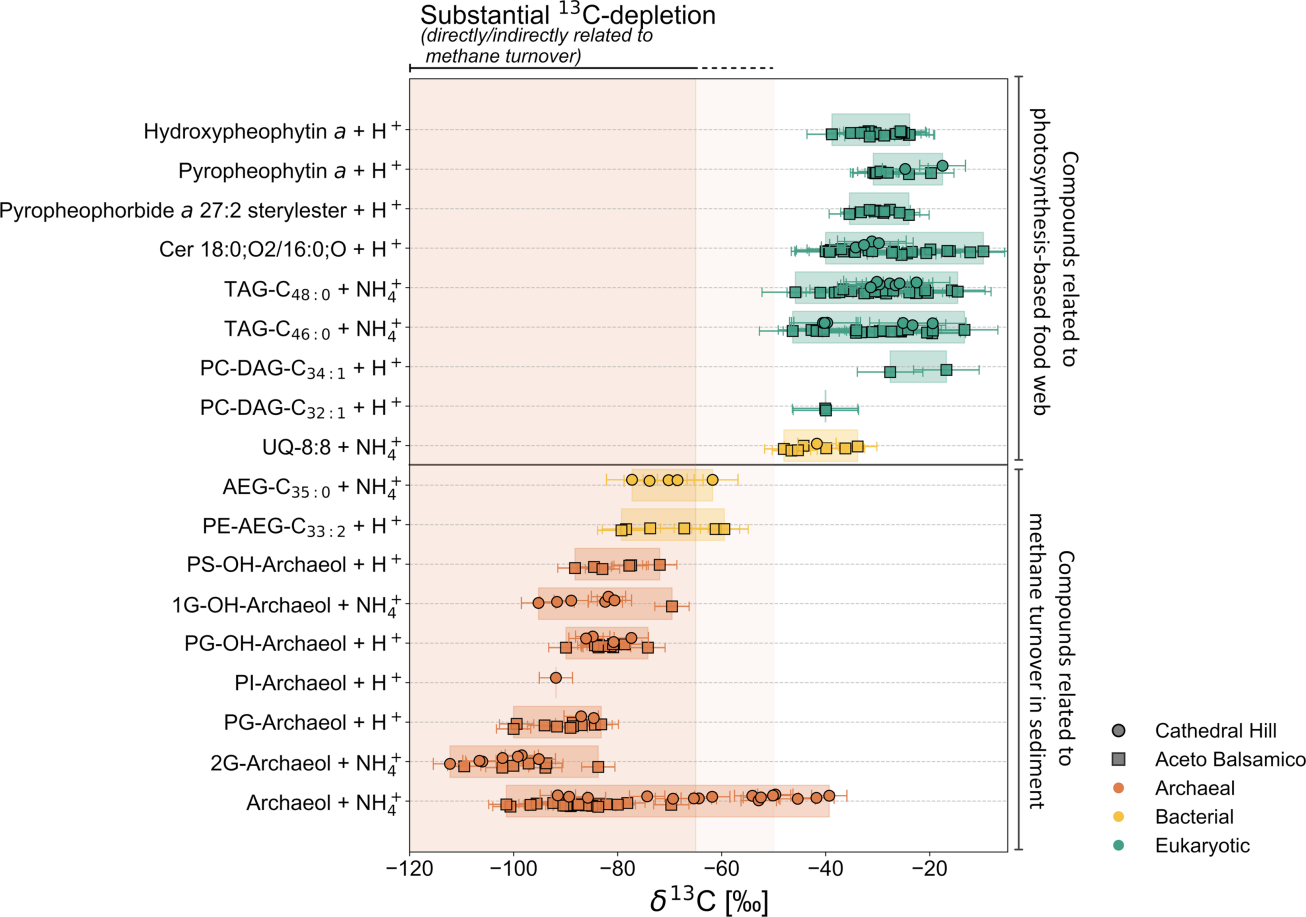
Horizontal strip plots of δ^13^C values in ‰, determined from M1/M0 ratios and following Equations (1) and (2), for various lipids, detected in sediment core 5000‐9 (Cathedral Hill, Guaymas Basin; circles) and 4992‐14 (Aceto Balsamico, Guaymas Basin; squares), including diglycosidic archaeol (2G‐Archaeol), phosphatidylglycerol archaeol (PG‐Archaeol), phosphatidylinositol archaeol (PI‐Archaeol), phosphatidylglycerol hydroxyarchaeol (PG‐OH‐Archaeol), monoglycosidic hydroxyarchaeol (1G‐OH‐Archaeol), phosphatidylserine hydroxyarchaeol (PS‐OH‐Archaeol); phosphatidylethanolamine acyl ether glycerol (PE‐AEG‐C_33:2_), acyletherglycerol (AEG‐C_35:0_); ubiquinone‐8:8 (UQ‐8:8), phosphatidylcholine diacylglycerol C_32:1_ (PC‐DAG‐C_32:1_), phosphatidylcholine diacylglycerol C_32:1_ (PC‐DAG‐C_32:1_), triacylglycerol‐C_46:0_ (TAG‐C_46:0_), triacylglycerol‐C_48:0_ (TAG‐C_48:0_), ceramide (Cer) 18;02/16;0, pyropheophorbide *a* 27:2 sterylester, pyropheophytin *a* and hydroxypheophytin *a*. Metabolites are categorized, based on whether a direct or indirect connection to methane turnover has been reported (bottom), versus those with no conceivable association to methane biogeochemistry (top). The most abundant adduct for each compound was used for δ^13^C calculation. Datapoints represent individual UHPLC/HRMS measurements following Wörmer et al. [31] for sediment samples along depth profiles (0–20 cm below seafloor at 2 cm intervals) that met the following conditions: M0 Peak area > 2 × 10^6^ arb.u. (see Figure 1), no coeluting peaks and no signs of detector oversaturation. The shaded region indicates the range of methanotrophic δ^13^C values reported in previous studies in environmental samples [5, 6, 7, 8, 9, 10, 11], with the gray shaded region highlighting the range (−65‰ to −50‰), where source attribution must be nuanced due to the higher uncertainty of this approach.

In the following, we demonstrate the potential of our proposed method as a high‐throughput approach for CSIA of a diverse pool of complex and intact compounds in raw lipid extracts. We selected multiple intact lipids extracted from the surface (0–20 cmbsf) of the Guaymas Basin sediment cores 5000‐9 (Cathedral Hill) and 4992‐14 (Aceto Balsamico) that are not amenable to GC‐based CSIA and calculated their δ^13^C values following Equations (1) and (2) (Figure 5). For each compound, the δ^2^H value used for correction was selected based on the predominant origin of the lipid (cf. Figure S1). First, we selected nine compounds where a connection to AOM can be confidently excluded on the basis of their chemotaxonomic classification. These include hydroxypheophytin *a*, pyropheophytin *a*, and pyropheophorbide *a* 27:2 steryl ester, all related to chlorophyll *a* derived from photosynthesizing organisms [69, 70, 71, 72]; a ceramide (Cer 18:0, O2/16:0,O) with various potential bacterial and eukaryotic sources, including fungi [73, 74, 75, 76]; triacylglycerols (TAG)‐C_48:0_ and C_46:0_ as storage lipids [77], likely originating from various microbial sources in the water column; and PC‐DAG‐C_34:1_ and PC‐DAG‐C_32:1_, commonly found in bacterial and eukaryotic phytoplankton and nitrifying and heterotrophic bacteria [78, 79, 80]. The electron carrier ubiquinone‐8:8 (UQ‐8:8) is primarily associated with diverse *Proteobacteria* [81, 82, 83], including some aerobic methanotrophic bacteria [84]. However, any contribution of UQ‐8:8 from aerobic methanotrophs is negligible due to the rapid oxygen depletion in the sediment [62, 85]. Although our approach is not suited for source assignment that relies on smaller isotopic differences (~ < 15‰), the observed δ^13^C values for these lipids, ranging from −11‰ to −44‰ (Figure 5), are broadly consistent with their chemotaxonomic classification as predominantly photosynthetic primary producers and/or secondary consumers utilizing photosynthetically derived organic matter [86, 87, 88, 89].

To contrast these comparably high δ^13^C values, we selected six IPLs widely recognized as potential biomarkers for ANME, specifically of the ANME‐2 cluster [90, 91], in methane‐rich environments. These include diglycosidic archaeol (2G‐archaeol), phosphatidylglycerol archaeol (PG‐archaeol), phosphatidylinositol archaeol (PI‐archaeol), phosphatidylglycerol hydroxyarchaeol (PG‐OH‐archaeol), monoglycosidic hydroxyarchaeol (1G‐OH‐archaeol), and phosphatidylserine hydroxyarchaeol (PS‐OH‐archaeol). Given the well‐documented presence of ANME in the surficial sediments of the Cathedral Hill and Aceto Balsamico area [62], an isotopically light signature of these lipids is expected. Indeed, all potential ANME‐related lipids are substantially ^13^C‐depleted with δ^13^C values ranging from −65‰ down to −114‰. These values fall within the δ^13^C range of archaeal lipids commonly found in sediments with high methane turnover (approximately −60‰ to −140‰) [5, 6, 7, 8, 9, 10, 11] (Figure 5). The chemotaxonomically less specific archaeol, which could be regarded as a degradation product from various intact archaeols, shows a larger range of δ^13^C values, indicating increased admixtures of other archaeal sources in some samples (cf. Figure 3).

Aside from the ^13^C‐depleted archaeal lipids, several bacterial ether lipids also showed considerable^13^C‐depletion. These include phosphatidylethanolamine acyletherglycerol C_33:2_ (PE‐AEG‐C_33:2_) and acyletherglycerol C_35:0_ (AEG‐C_35:0_), whose δ^13^C values remain below −60‰ (Figure 5). In various marine environments affected by AOM processes, PE‐AEG lipids are often found to be associated with mesophilic or thermophilic sulfate‐reducing bacteria (SRB) that are considered to be syntrophic partner bacteria in AOM [90, 92, 93, 94]. In hydrothermal sediments of the Guaymas Basin, SRB commonly occur in association with sulfate‐dependent AOM [61, 62, 63]. The observed ^13^C‐depletion in PE‐AEG‐C_33:2_ and PE‐AEG‐C_35:0_ in our samples indicates that the indirect incorporation of methane‐derived carbon contributes to the ^13^C‐depleted nature of the corresponding lipid biomarkers as observed elsewhere [16].

The clear separation of isotopically light AOM‐associated core and intact lipids from comparatively ^13^C‐enriched compounds with no connection to methane biogeochemistry presented in Figure 5 underscores the utility of our proposed method for δ^13^C analysis of intact molecules in complex samples, which intends to identify the distinctly ^13^C‐depleted molecular signals indicative of the assimilation of methane‐derived carbon. We emphasize that our proposed approach is not designed to achieve per‐mille level precision and accuracy, and the analytical uncertainty, the uncertainty introduced by the approximated δ^2^H value, and potential adduct‐dependent offset (e.g., H^+^ and NH₄^+^) must be acknowledged as detailed in Section 3.2. In particular, when δ^13^C values fall in a transitional range of ~−50‰ to −65‰, a straightforward distinction of lipids from methane‐oxidizing and methanogenic archaea may be difficult [16].

There is broad potential for applying the isotopologue‐based approach for δ^13^C analysis in lipidomics for targeting complex and intact lipids not amenable to traditional GC‐based CSIA. Particularly interesting target compounds include glycerol dialkyl glycerol tetraethers (GDGTs), which are frequently associated with ANME‐1 in methane‐rich environments [90, 91]. Although the GDGT signals in the current dataset did not meet the required criteria (peak areas below the set threshold and minor coelution of ring‐bearing GDGTs) for robust δ^13^C calculation, our approach is applicable for δ^13^C analysis of GDGT‐based lipids in other datasets where the required criteria are met. Beyond the identification of isotopic signatures related to methane turnover, our approach may also be applied to experiments involving ^13^C‐labeled substrates. The ^13^C enrichment typically observed in lipids during labeling experiments far exceeds the natural isotopic variability, making our approach highly suitable for tracing compound‐specific^13^C‐label incorporation.

## 4 Conclusions

We demonstrated that the M1/M0 ratios acquired from UHPLC/HRMS analysis of sedimentary lipid extracts capture natural isotopic variations related to extreme ^13^C‐depletion in lipids associated with microbial methane oxidation and can be utilized to calculate approximate δ^13^C values according to Equations (1) and (2). To produce reliable δ^13^C values, it is essential to determine the optimal intensity range for a given MS system and LC separation protocol in use while ensuring that coeluting peaks are avoided. When these criteria are met, this technique offers a promising approach for determining the stable carbon isotopic compositions of a wide range of microbial lipids that were previously inaccessible via GC/irMS. We emphasize that this approach is currently focused exclusively on identifying large isotopic deviations characteristic of lipids related to high methane turnover and is not optimized for more fine‐scale isotopic fingerprinting. Other future applications include stable isotope probing experiments targeting lipids, where the accuracy and precision achieved here are typically fully satisfactory for the quantification of label uptake. In conclusion, our proposed approach enables the analysis of a wide spectrum of nonvolatile, chemotaxonomically specific biomolecules with the aim of detecting microbial methane turnover in complex environmental samples.

## Author Contributions

**Janina Groninga:** conceptualization, writing – original draft, investigation, visualization, writing – review and editing, formal analysis. **Julius Lipp:** conceptualization, writing – review and editing, methodology, resources. **Min Song:** writing – review and editing, resources. **Kai‐Uwe Hinrichs:** conceptualization, supervision, resources, writing – review and editing.

## Conflicts of Interest

The authors declare no conflicts of interest.

## Supporting information


**Table S1:** Three examples showing the influence of different δ^15^N values on δ^13^C determination based on Equations (1) and (2), assuming a fixed δ^2^H value of −234‰. M1/M0 ratio for ammoniated archaeolstd (C43H88O3 + NH4^+^) was adjusted using an assumed δ^15^N value of −30‰, 0‰, and 30‰ relative to atmospheric N2 to account for potential variation in the nitrogen isotopic composition.
**Figure S1:** Variability of reported δ^2^H values (‰ vs. VSMOW) in lipids derived from environmental matrices, categorized by the inferred biological origin. Lipids were grouped into archaeal, bacterial, and eukaryotic based on chemotaxonomy. **Archaeal lipids:** Archaeol, hydroxyarchaeol, biphytanes. **Bacterial lipids:** Methyl‐branched fatty acids, odd‐chain fatty acids (C_15_, C_17_, C_19_), cyclopropyl fatty acids, and hopanols. **Eukaryotic lipids:** Even‐chain short‐chain fatty acids (C_14_, C_16_, C_18_), polyunsaturated fatty acids, sterols, phytol, and phytol derivatives. Minimum, maximum, and midpoint δ^2^H values are shown for each group. δ^2^H data were collected from the following studies: Sessions et al. [34]; Li et al. [43]; Jones et al. [42]; Naraoka et al. [46]; Kaneko et al. [35]; Osburn et al. [45]; Wegener et al. [48]; Dawson et al. [47]; Kellerman et al. [44]; Heinzelmann et al. [39, 41].
**Figure S2:** Relationship between the on‐column of archaeol standard injections (in ng) and δ13C value. The δ^13^C values were calculated for the [M + NH₄]⁺ adduct and all measurements were conducted using reversed phase‐UHPLC/ESI/qToF/MS following the method of Wörmer et al. [31]. The dashed line indicates the δ^13^C value of the archaeol standard determined by EA‐IRMS (−33.3‰).

## Data Availability

The data that support the findings of this study are available from the corresponding author upon request. Georeferenced data can be accessed via the PANGAEA data repository.
