# Supplementary material for: Isotopologue Ratios Identify 13C‐Depleted Biomarkers in Environmental Samples Impacted by Methane Turnover
Source: Rapid Commun Mass Spectrom. 2025 Aug 5;39(22):e10118. doi: 10.1002/rcm.10118 (PMC12322946; doi:10.1002/rcm.10118)
Supplement: Supplementary file 1 — Table S1: Three examples showing the influence of different δ15N values on δ13C determination based on Equations (1) and (2), assuming a fixed δ2H value of −234‰. M1/M0 ratio for ammoniated archaeolstd (C43H88O3 + NH4+) was adjusted using an assumed δ15N value of −30‰, 0‰, and 30‰ relative to atmospheric N2 to account for potential variation in the nitrogen isotopic composition. Figure S1: Variability of reported δ2H values (‰ vs. VSMOW) in lipids derived from environmental matrices, categorized by the inferred biological origin. Lipids were grouped into archaeal, bacterial, and eukaryotic based on chemotaxonomy. Archaeal lipids: Archaeol, hydroxyarchaeol, biphytanes. Bacterial lipids: Methyl‐branched fatty acids, odd‐chain fatty acids (C15, C17, C19), cyclopropyl fatty acids, and hopanols. Eukaryotic lipids: Even‐chain short‐chain fatty acids (C14, C16, C18), polyunsaturated fatty acids, sterols, phytol, and phytol derivatives. Minimum, maximum, and midpoint δ2H values are shown for each group. δ2H data were collected from the following studies: Sessions et al. [34]; Li et al. [43]; Jones et al. [42]; Naraoka et al. [46]; Kaneko et al. [35]; Osburn et al. [45]; Wegener et al. [48]; Dawson et al. [47]; Kellerman et al. [44]; Heinzelmann et al. [39, 41]. Figure S2: Relationship between the on‐column of archaeol standard injections (in ng) and δ13C value. The δ13C values were calculated for the [M + NH₄]⁺ adduct and all measurements were conducted using reversed phase‐UHPLC/ESI/qToF/MS following the method of Wörmer et al. [31]. The dashed line indicates the δ13C value of the archaeol standard determined by EA‐IRMS (−33.3‰). [file RCM-39-e10118-s001.pdf]

Supplementary Material

Table S1. Three examples showing the influence of different $\delta^{15}N$ values on $\delta^{13}C$ determination based on Eqs. 1 and 2, assuming a fixed $\delta^{2}H$ value of -234‰. M1/M0 ratio for ammoniated archaeol$_{std}$ ($C_{43}H_{88}O_3$ + NH_4^+) was adjusted using an assumed $\delta^{15}N$ value of -30‰, 0‰, and 30‰ relative to atmospheric N_2 to account for potential variation in the nitrogen isotopic composition.

**$C_{43}H_{88}O_3$ +NH_4^+**			**Influence on calculated $\delta^{13}C$ values**	
M0 area [arb.u.]	M1 area [arb.u.]	M1/M0	$\delta^{15}N$ [‰]	$\delta^{13}C$ ($\delta^{2}H$ = -234‰) [‰]
			30	-31.4
4308658	2074659	0.482	0	-31.2
			-30	-31.0

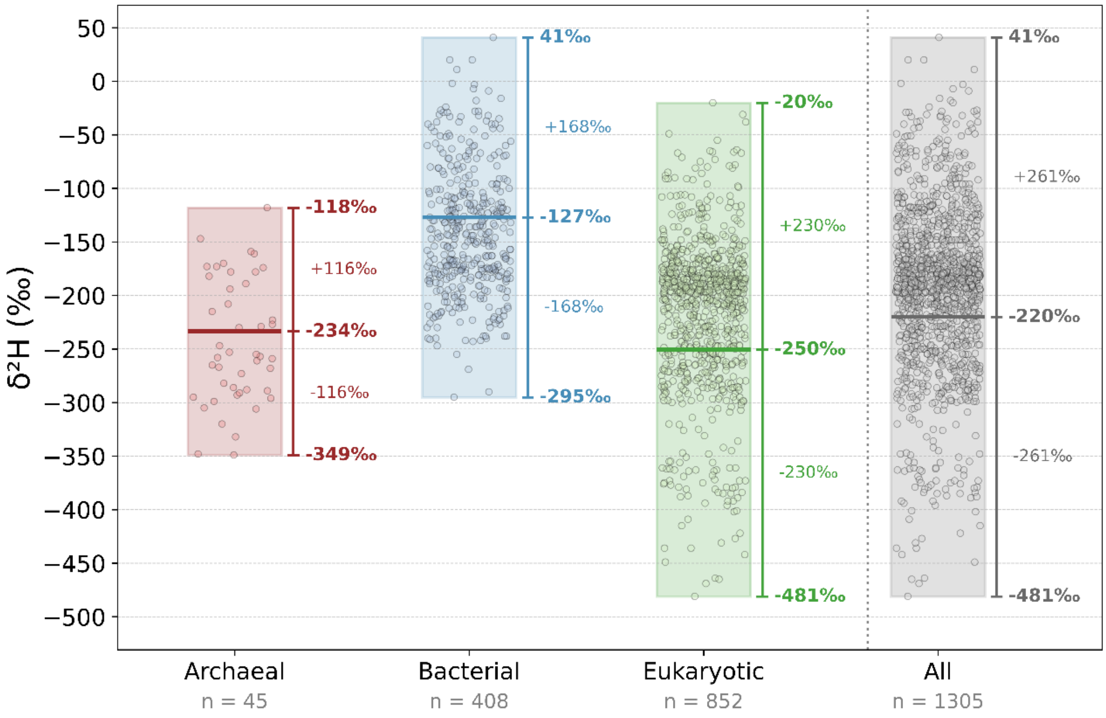

Figure S1.Variability of reported $\delta^{2}H$ values (‰ vs. VSMOW) in lipids derived from environmental matrices, categorized by the inferred biological origin. Lipids were grouped into archaeal, bacterial, and eukaryotic based on chemotaxonomy. **Archaeal lipids:** Archaeol, hydroxyarchaeol, biphytanes. **Bacterial lipids:** Methyl-branched fatty acids, odd-chain fatty acids (C_{15}, C_{17}, C_{19}) cyclopropyl fatty acids, and hopanols. **Eukaryotic lipids:** Even-chain short-chain fatty acids (C_{14}, C_{16}, C_{18}), polyunsaturated fatty acids, sterols, phytol, and phytol derivatives. Minimum, maximum, and midpoint $\delta^{2}H$ values are shown for each group. $\delta^{2}H$ data was collected from the following studies: Sessions et al.[34]; Li et al.[43]; Jones et al.[42]; Naraoka et al.[46]; Kaneko et al.[35]; Osburn et al.[45]; Wegener et al.[48]; Dawson et al.[47]; Kellerman et al.[44]; Heinzelmann et al.[39,41].

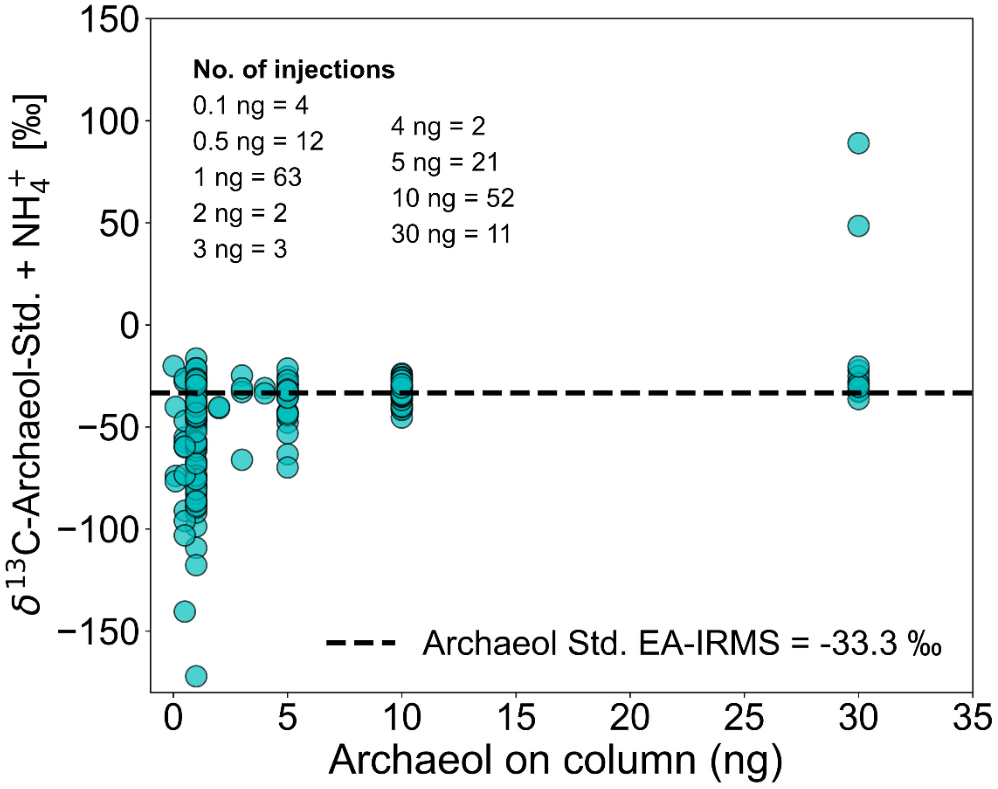

Figure S2. Relationship between the on-column of archaeol standard injections (in ng) and $\delta^{13}C$ value. The $\delta^{13}C$ values were calculated for the $[M + NH_4]^+$ adduct and all measurements were conducted using reversed phase-UHPLC/ESI/qToF/MS following the method of Wörmer et al.[31]. The dashed line indicates the $\delta^{13}C$ value of the archaeol standard determined by EA-IRMS (−33.3 ‰).

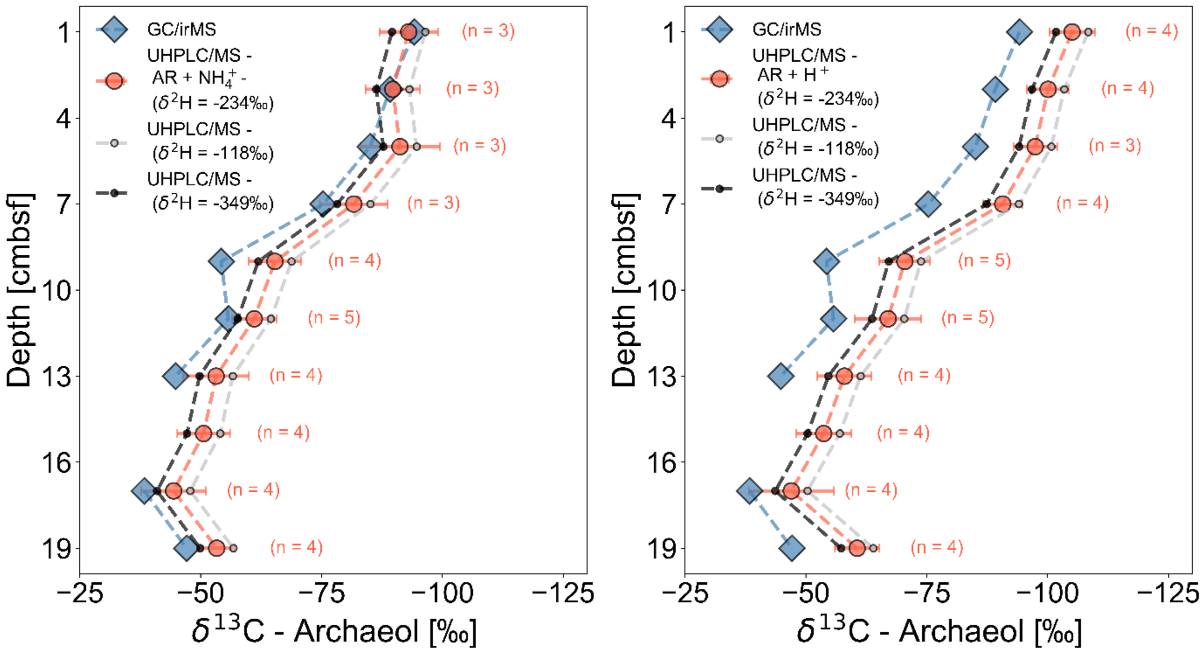

Figure S3. Comparison of δ^{13}C values [‰] obtained from GC/irMS (blue diamonds) and reversed-phase UHPLC/ESI/HRMS following Eqs. 1 and 2 for archaeol + NH_4^+ and archaeol + H^+ (orange circles) across sediment core 5000-9 (Cathedral Hill). δ^{13}C values were calculated according to Eqs. 1 and 2 using a fixed δ^2H value of -234‰. Additional δ^{13}C calculations using δ^2H values of −118‰ (grey) and −349‰ (black), respectively, illustrate the isotopic shift introduced via δ^2H variability (cf. Figure S1). Error bars reflect the propagated error, combining δ^2H-introduced uncertainty and the analytical standard deviation across multiple independent UHPLC/HRMS measurements, with n=X indicating the number of runs, fulfilling the following criteria: Only peaks with an integrated M0 area above 2x10^6 arb.u. and 5x10^6 arb.u. were included, depending on the applied LC separation methods after Wörmer et al.[31] and Zhu et al.[32], respectively. Peaks showing signs of coelution, or oversaturation were excluded.

27
